# *Aspergillus terreus* Antibody Serosurveillance in Tyrol: A Population-Based, Cross-Sectional Study of a Healthy Population

**DOI:** 10.3390/jof9101008

**Published:** 2023-10-12

**Authors:** Stephan Steixner, Roya Vahedi Shahandashti, Anita Siller, Hanno Ulmer, Norbert Reider, Harald Schennach, Cornelia Lass-Flörl

**Affiliations:** 1 Institute of Hygiene and Medical Microbiology, Medical University of Innsbruck, 6020 Innsbruck, Tirol, Austria; stephan.steixner@i-med.ac.at (S.S.); roya.vahedi@i-med.ac.at (R.V.S.); 2 Central Institute for Blood Transfusion and Immunology, Tirol Kliniken GmbH, 6020 Innsbruck, Tirol, Austria; anita.siller@tirol-kliniken.at (A.S.); harald.schennach@tirol-kliniken.at (H.S.); 3 Department of Medical Statistics, Informatics and Health Economics, Medical University of Innsbruck, 6020 Innsbruck, Tirol, Austria; hanno.ulmer@i-med.ac.at; 4 Department of Dermatology, Venereology and Allergology, Medical University of Innsbruck, 6020 Innsbruck, Tirol, Austria; norbert.reider@i-med.ac.at

**Keywords:** *Aspergillus terreus*, IgG antibodies, serosurveillance, population-based, cross-sectional study, emerging mold

## Abstract

The rare, but emerging mold *Aspergillus terreus* is an important pathogen in some geographical areas, like Tyrol (Austria) and Houston (Texas). The reason for this high prevalence is unknown. The present serosurveillance study aimed to evaluate the trends in levels of *A. terreus*-specific IgG antibodies in various regions of Tyrol and to compare the results to the environmental spread of *A. terreus* in Tyrol. Therefore, 1058 serum samples from healthy blood donors were evaluated. Data revealed a significant difference between the Tyrolean Upland and Lowland. Moreover, female participants had higher *A. terreus* IgG antibody levels than male participants. The differences found in our study are consistent with the distributional differences in environmental and clinical samples described in previous studies, supporting that *A. terreus* IgG antibody levels reflect the environmental epidemiology of *A. terreus* in Tyrol.

## 1. Introduction

*Aspergillus* species are among the most prevalent saprophytic filamentous fungi [1,2], manifesting a broad range of diseases, namely invasive aspergillosis (IA), allergic bronchopulmonary aspergillosis (ABPA) and chronic pulmonary aspergillosis (CPA) [3,4]. Aspergillosis is a life-threatening infection characterized by high mortality rates ranging up to 40–50%, mainly occurring in immunocompromised patients [5].

The *Aspergillus fumigatus* species complex is the most common etiological agent within the genus of *Aspergillus*, followed by less common species complexes such as *A. flavus*, *A. niger* and *A. terreus* [3,6]. Depending on the geographical region, *A. terreus* can be either the third or the fourth most common etiological agent of IA [7]. Areas with a higher *A. terreus* abundance are Tyrol (Austria), Houston (Texas), Spain and Israel [8,9].

The significance of infections caused by less common species such as *A. terreus* (contributing to 4–5.2% of IA cases worldwide) has increased as the epidemiology of invasive fungal infections (IFIs) continues to change, with a wider range of patients appearing to be at risk [8,10,11]. In addition to a changing epidemiology and an expanding at-risk population, *A. terreus* presents clinical challenges due to its unique characteristics. Infections from *A. terreus* are represented with a high tendency for dissemination and a mortality up to 70% in IA [3,12]. Moreover, the natural lower susceptibility of *A. terreus* to amphotericin B and increasing azole resistance, even in some less common species of section Terrei, are of particular concern, which may result in complications in patient treatment [10,13,14,15]. Furthermore, in Tyrol (Austria), *A. terreus* plays an important role due to the significantly higher number of clinical cases when compared globally [3,9,16]. Since 1994, infections caused by *A. terreus* have been detected at the University Hospital Innsbruck, which were mainly related to construction activities [9].

*A. terreus* has a significant, uneven environmental distribution between the Tyrolean Upland (West) and Lowland (East), with the capital city of Innsbruck located in the middle of Tyrol [17]. Dietl et al. found that 61.9% of environmental samples came from the Tyrolean Lowland, whereas only 11.6% of the Tyrolean Upland samples resulted in the isolation of *A. terreus* [17]. Furthermore, the distribution of patients with proven infection or colonization with *A. terreus* at the Institute of Hygiene and Medical Microbiology of the Medical University of Innsbruck correlated well with these findings (Upland: 9.43%; Lowland: 56.6%) [17]. Dietl et al. have evaluated several environmental, economic and microclimatic variables, like temperature, vegetation, population density and wind, concluding that none of these factors resulted in significant differences within the area of Tyrol [17].

As *Aspergillus* spores are constantly found in the air and human respiratory tract, antibodies against an invading or colonizing pathogen are formed both in healthy as well as in immunocompromised humans [4,18]. Mold-specific immunoglobulin G (IgG) antibodies are widely used as biomarkers in research of environmental molds [19]. Further, it has been shown that levels of mold-specific IgG antibodies reflect exposure to the specific pathogen [20]. Serosurveillance is known as an effective tool for measuring infection prevalence and incidence [21] and could give valuable insights into the differences among the environmental spread of *A. terreus* in Tyrol.

The aim of this study was to assess the trends in *A. terreus*-specific IgG antibody levels in a serosurveillance analysis of a healthy population in Tyrol and to determine whether the data reflect the different distribution of *A. terreus* between the Tyrolean Upland and Lowland.

## 2. Materials and Methods

### 2.1. Ethics Statement

Serum samples were collected from Tyrolean blood donors for this population-based, cross-sectional study. The Human Ethics Committee of the Medical University of Innsbruck evaluated and approved the protocol for this study in 2022 (EK No. 1284/2022).

### 2.2. Study Population

Between January 2023 and April 2023, a total of 1058 blood samples were collected—362 samples from blood donors in the Tyrolean Upland and 696 samples from blood donors in the Tyrolean Lowland—in cooperation with the Central Institute for Blood Transfusion and Immunology of the University Hospital Innsbruck and the Tyrolean Red Cross blood donation service. Samples were collected as part of routine infection serology testing. This study included only participants registered as residents of Tyrol.

### 2.3. Investigation of A. terreus IgG Antibody Levels in Serum Samples of a Healthy Tyrolean Population

Samples from participants with signed consent for sample use for scientific purposes were collected and further processed by an ETI-Max 3000 (DiaSorin S.p.A., Centralino, Italy) to obtain 950 µL of serum. Samples were stored at −80 °C prior to use.

All serum samples were analyzed on a PhadiaTM 250 laboratory system (Thermo Fisher Scientific Inc., Waltham, MA, USA) using an ImmunoCap allergen m36 for *A. terreus* (Thermo Fisher Scientific Inc., Waltham, MA, USA). Specimens were analyzed according to the manufacturer’s protocol. As our aim was to investigate serum antibody levels in a healthy population without any bias on diseases, we did not use a positive cut-off value. All antibody levels above the detection limit (0.02 mg/L) were enrolled.

### 2.4. Statistical Analysis

Data were checked for normality and homogeneity prior to analysis. The statistical analysis was performed using GraphPad Prism9 (GraphPad Software, Boston, MA, USA). A Mann–Whitney U test was used to compare the data collected between the Tyrolean Upland and Lowland.

Data for the comparison among the different blood donation sites were analyzed using a Kruskal–Wallis test with Dunn’s correction for multiple comparisons. Sex-specific data for the Tyrolean Upland and Lowland were analyzed via a Mann–Whitney U test and two-way analysis of variance (ANOVA) with Bonferroni’s correction for multiple comparisons. IgG values are presented as mean ± SD. A *p* value of less than 0.05 (*p* < 0.05) was considered statistically significant.

## 3. Results

### 3.1. Patient Population Data

In total, 1058 serum samples from blood donors all over Tyrol were collected and analyzed for their levels of IgG antibodies against *A. terreus*. A total of 1046 samples were used from this 1058 sample set, with 355 samples coming from the Tyrolean Upland and 691 from the Tyrolean Lowland. Twelve samples were excluded from the study because they were not registered as residents of Tyrol.

This study involved 446 female (42.64%) and 600 male (57.36%) blood donors (Upland: 143/212; Lowland: 303/388). The study population showed a mean age of 46.58 ± 13.64 years (Upland/Lowland: 46.13 ± 13.77/46.81 ± 13.58; female/male: 44.95 ± 13.82/47.79 ± 13.39), ranging from 18 to 70 years.

### 3.2. Serosurveillance of IgG Antibody Levels in the Tyrolean Upland and Lowland

**Figure 1:**
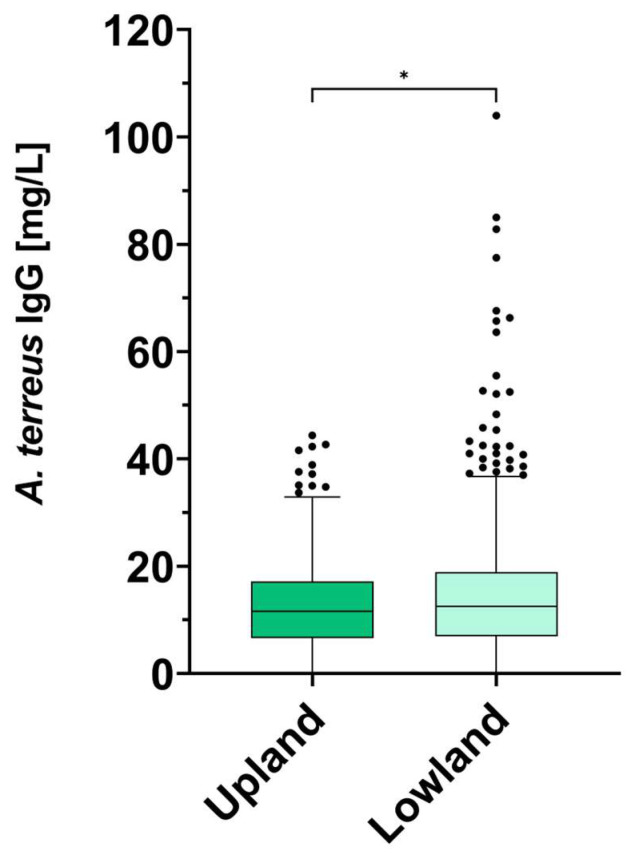
Differences in *A. terreus* antibody levels (mg/L) between the Tyrolean Upland and Lowland. The measurements are depicted as a box-and-whiskers Tukey plot. The detection limit was 0.02 mg/L. A Mann–Whitney U test resulted in a *p* value of 0.0468 (* *p* < 0.05).

The results of the comparison of *A. terreus* IgG antibody levels between the Tyrolean Upland and Lowland showed a statistical difference (*p* = 0.0468) (Figure 1). Inhabitants of the Tyrolean Upland had lower *A. terreus* IgG antibody levels (12.90 ± 8.53 mg/L) than inhabitants of the Tyrolean Lowland (14.88 ± 11.76 mg/L) (Figure 1). Only 15 study participants from the Tyrolean Upland and 10 participants from the Tyrolean Lowland showed *A. terreus* IgG antibody levels lower than the detection limit. These values were therefore set to the detection limit of 0.02 mg/L for the statistics.

**Figure 2:**
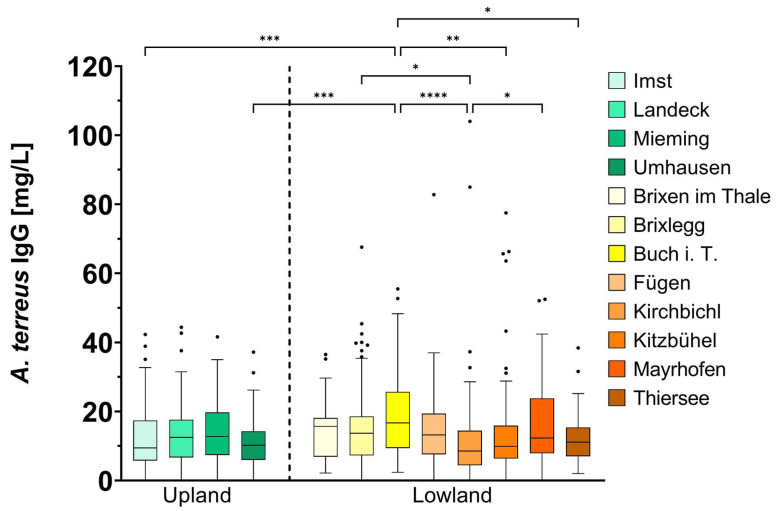
Differences in *A. terreus* antibody levels (mg/L) grouped according to blood donation sites. The measurements are depicted as a box-and-whiskers Tukey plot. The detection limit was 0.02 mg/L. A Kruskal–Wallis test with Dunn’s correction for multiple comparison resulted in *p* < 0.05 for several blood donation sites (**** *p <* 0.0001; *** *p* < 0.001; ** *p* < 0.01; * *p* < 0.05).

When samples were grouped into the different blood donation sites, several statistically significant results could be found (Figure 2). Regarding the Tyrolean Upland, no significant differences in the levels of *A. terreus* IgG antibodies between the separate donation sites could be observed. However, there was a tendency for participants from Umhausen to have the lowest and participants from Mieming to have the highest *A. terreus* IgG antibody levels within the Tyrolean Upland. Concerning the Tyrolean Lowland, *A. terreus* IgG antibody levels were significantly higher among donors in Buch in Tirol (Buch i. T., 18.59 ± 11.51 mg/L) when compared to different locations in the Tyrolean Lowland, specifically Kirchbichl, Kitzbühel and Thiersee. Concerning the whole of Tyrol, blood donors in the Tyrolean Upland from Imst and Umhausen showed significantly lower antibody levels than donors from Buch i. T. in the Tyrolean Lowland.

**Figure 3:**
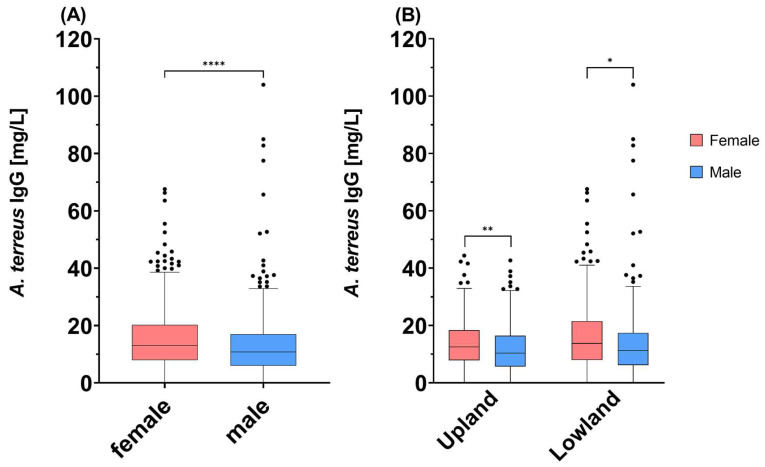
Differences in *A. terreus* IgG antibody levels between (**A**) female and male study participants; (**B**) female and male study participants grouped into the Tyrolean Upland and Lowland. Measurements are depicted as a box-and-whiskers Tukey plot. The detection limit was 0.02 mg/L. Statistical differences were determined via (a) a Mann–Whitney U test; (b) two-way ANOVA with Bonferroni’s correction for multiple comparisons. Statistical analysis resulted in a *p* value of (a) *p* < 0.0001; (b) Upland: *p* = 0.0049, Lowland: *p* = 0.0177 (**** *p* < 0.0001; ** *p* < 0.01; * *p* < 0.05).

With the influence of sex taken into consideration, female study participants showed significantly higher *A. terreus* IgG antibody levels than male participants (*p* < 0.0001) (Figure 3A). Female participants had *A. terreus* IgG antibody levels of 15.77 ± 10.75 mg/L, whereas male participants showed levels of 13.05 ± 10.72 mg/L. Statistical significance was sustained for both female and male groups when separated into Tyrolean Upland and Lowland (Upland: *p* = 0.0049; Lowland: *p* = 0.0177) (Figure 3B).

## 4. Discussion

An increasing number of vulnerable individuals is making invasive fungal infections (IFIs) more prevalent [22]. IFIs result in approximately 1.5 million deaths globally each year, with 70% resulting from an infection with fungi of the genera *Aspergillus*, *Candida*, *Cryptococcus* and *Histoplasma* [23]. Aspergillosis, usually caused by *A. fumigatus*, can lead to severe pulmonary infections [3,4,6].

Despite the global dominance of *A. fumigatus*, *A. terreus* shows a higher infection rate in some global areas, such as Tyrol, Austria [8,9,16]. A prospective international study with respect to *A. terreus* showed a global prevalence of 5.2% of mold infections [8]. *A. terreus* is restricted geographically to some areas with higher infection rates, like Tyrol (Austria), Spain, Israel and Houston (Texas) [9,16]. There is no clear explanation for this distributional difference, which requires further investigation [13].

*A. terreus* is known to be a unique representative of the *Aspergillus* genus because of its low susceptibility to amphotericin B, which makes the treatment of infections from this pathogen challenging [9,24,25,26,27]. Furthermore, *A. terreus* is commonly used as a producer of natural statins, such as compactin, mevastatin and lovastatin or itaconic acid [28].

In a study by Dietl et al., 5.4% of environmental samples (soil, air, plant material, etc.) were culture-positive for *A. terreus*. These results indicate an increased environmental burden with respect to *A. terreus* when compared to other regions like Cologne and Madrid, with only 0.2% and 0.5% of samples, respectively [17,18,29]. The study by Dietl et al. further revealed that, even within Tyrol, an uneven distribution can be observed, with the Tyrolean Upland (11.6%) resulting in a lower prevalence than the Tyrolean Lowland (61.9%) [17]. Currently, there are no known factors of significant difference between the two regions of Tyrol that could result in the different environmental spread of *A. terreus.* In their study, Dietl et al. evaluated several environmental, economic and climatic variables, such as temperature, vegetation, altitude, population density, traffic and wind, and concluded that all of these factors are comparable within the area of Tyrol [17]. Other possible factors, like microclimate, topography, geographical diversity, humidity, agricultural use of antifungals and socio-economic factors, need to be analyzed further in subsequent studies.

In our project, we used serum samples from healthy blood donors in Tyrol. The study population consisted of 446 female and 600 male participants and showed a mean age of 47 ± 14, ranging from 18 to 70 years. Some studies suggest that immunosenescence has no impact on the quantity of antibody production, but the elderly show lower production of specific antibodies against invading pathogens [30,31]. Our results are somewhat contradictory as the highest levels of *A. terreus* IgG antibodies derived from study participants who were 50 years of age or older. A study including 100 healthy Ugandan blood donors (mean age of 19 years) displayed a median *Aspergillus*-specific IgG level of 5 mg/L, with no participants having higher than 40 mg/L [32]. The present study showed a mean *A. terreus* IgG antibody level of 14.29 ± 11.14 mg/L (Upland: 12.90 ± 8.53 mg/L; Lowland: 14.88 ± 11.76 mg/L); comparable data and literature on *A. terreus* are largely absent. Lee et al. showed that in healthy blood donors from Taiwan, mean IgG antibody levels for *A. fumigatus* and *A. niger* were 28.6 mg/L and 20.3 mg/L [33]. In their study, age, female sex, tuberculosis and rural residency were associated with *A.* fumigatus-specific IgG >50 mg/L; as well, a lower risk of cancer was associated with an IgG >50 mg/L. The prevalence of specific antibodies seems to be widely influenced.

Our data revealed that blood donors from the Tyrolean Upland have significantly lower *A. terreus* IgG antibody levels than study participants from the Tyrolean Lowland (Figure 1). As antibodies against an invading or colonizing pathogen are formed both in healthy as well as in immunocompromised humans, IgG antibodies can be used as biomarkers in research [4,19]. Our results imply that humans living in an area of high *A. terreus* prevalence, like the Tyrolean Lowland, also develop high levels of IgG antibodies against this mold. The serosurveillance of *A. terreus* IgG antibodies further shows that, even within the Tyrolean Lowland, differences can be observed. Blood donors from Buch i. T. had significantly higher *A. terreus* IgG antibody levels than blood donors from several other donation sites. These findings are in agreement with a previous study [17]. Both Buch i. T. as well as Mayrhofen are located in the district of Schwaz, which is known to have a higher prevalence of *A. terreus* in the environment, followed by the district of Kufstein [17].

Besides the significant difference between the Tyrolean Upland and Lowland, a difference in *A. terreus* IgG antibody levels between female and male participants could be observed (Figure 3A). Female blood donors showed higher levels of IgG antibodies against *A. terreus*. So far, this sex-specific difference is novel for *A. terreus* and could be related to the fact that adult females tend to have higher antibody responses than males, as has been mentioned for different species [34,35,36]. As shown by Klein and Flanagan, females have a higher B-cell count, higher immunoglobulin levels and a better antibody response than males, which could be associated with female hormones or the X chromosome [36]. Our results are also in concordance with findings from Lee et al., showing an odds ratio of 1.49 (95% CI: 1.14–1.93) for antibody formation in females [33]. Significant differences between female and male participants persisted even when they were split into the Tyrolean Upland and Lowland (Figure 3B).

## 5. Conclusions

*A. terreus* plays an important role in Tyrol (Austria), and our results are in line with the fact that this species has a higher abundance in the Tyrolean Lowland. The location of Buch i. T. and Mayrhofen in the Tyrolean Lowland can be identified as possible hotspots. The reasons for the high occurrence of *A. terreus* in the Tyrolean Lowland needs further investigation.

## Data Availability

All data are contained within the article.

## References

[1] Chen B., Qian G., Yang Z., Zhang N., Jiang Y., Li D., Li R., Shi D. (2023). Virulence capacity of different *Aspergillus* species from invasive pulmonary aspergillosis. Front. Immunol..

[2] Visagie C.M., Houbraken J. (2020). Updating the taxonomy of *Aspergillus* in South Africa. Stud. Mycol..

[3] Lass-Flörl C., Dietl A.M., Kontoyiannis D.P., Brock M. (2021). *Aspergillus terreus* species complex. Clin. Microbiol. Rev..

[4] Page I.D., Richardson M., Denning D.W. (2015). Antibody testing in aspergillosis—Quo vadis?. Med. Mycol..

[5] Latge J.P., Chamilos G. (2019). *Aspergillus fumigatus* and Aspergillosis in 2019. Clin. Microbiol. Rev..

[6] Lass-Flörl C. (2018). Treatment of infections due to *Aspergillus terreus* species complex. J. Fungi.

[7] Neal C.O., Richardson A.O., Hurst S.F., Tortorano A.M., Viviani M.A., Stevens D.A., Balajee S.A. (2011). Global population structure of *Aspergillus terreus* inferred by ISSR typing reveals geographical subclustering. BMC Microbiol..

[8] Risslegger B., Zoran T., Lackner M., Aigner M., Sanchez-Reus F., Rezusta A., Chowdhary A., Taj-Aldeen S.J., Arendrup M.C., Oliveri S. (2017). A prospective international *Aspergillus terreus* survey: An EFISG, ISHAM and ECMM joint study. Clin. Microbiol. Infect..

[9] Lass-Flörl C., Griff K., Mayr A., Petzer A., Gastl G., Bonatti H., Freund M., Kropshofer G., Dierich M.P., Nachbaur D. (2005). Epidemiology and outcome of infections due to *Aspergillus terreus*: 10-year single centre experience. Br. J. Haematol..

[10] Vahedi-Shahandashti R., Dietl A.M., Binder U., Nagl M., Würzner R., Lass-Flörl C. (2022). *Aspergillus terreus* and the interplay with amphotericin B: From resistance to tolerance?. Antimicrob. Agents Chemother..

[11] Lass-Flörl C., Cuenca-Estrella M. (2017). Changes in the epidemiological landscape of invasive mould infections and disease. J. Antimicrob. Chemother..

[12] Tritz D.M., Woods G.L. (1993). Fatal disseminated infection with *Aspergillus terreus* in immunocompromised hosts. Clin. Infect. Dis..

[13] Vahedi Shahandashti R., Lass-Flörl C. (2019). Antifungal resistance in *Aspergillus terreus*: A current scenario. Fungal Genet. Biol..

[14] Vahedi-Shahandashti R., Hahn L., Houbraken J., Lass-Flörl C. (2023). *Aspergillus* section *Terrei* and antifungals: From broth to agar-based susceptibility testing methods. J. Fungi.

[15] Zoran T., Sartori B., Sappl L., Aigner M., Sanchez-Reus F., Rezusta A., Chowdhary A., Taj-Aldeen S.J., Arendrup M.C., Oliveri S. (2018). Azole-resistance in *Aspergillus terreus* and related species: An emerging problem or a rare phenomenon?. Front. Microbiol..

[16] Lass-Flörl C., Grif K., Kontoyiannis D.P. (2007). Molecular typing of *Aspergillus terreus* isolates collected in Houston, Texas, and Innsbruck, Austria: Evidence of great genetic diversity. J. Clin. Microbiol..

[17] Dietl A.M., Vahedi-Shahandashti R., Kandelbauer C., Kraak B., Lackner M., Houbraken J., Lass-Flörl C. (2021). The environmental spread of *Aspergillus terreus* in Tyrol, Austria. Microorganisms.

[18] Guinea J., Pelaez T., Alcala L., Bouza E. (2006). Outdoor environmental levels of *Aspergillus* spp. conidia over a wide geographical area. Med. Mycol..

[19] Rydjord B., Hetland G., Wiker H.G. (2005). Immunoglobulin G antibodies against environmental moulds in a Norwegian healthy population shows a bimodal distribution for *Aspergillus versicolor*. Scand. J. Immunol..

[20] Makkonen K., Viitala K.I., Parkkila S., Niemela O. (2001). Serum IgG and IgE antibodies against mold-derived antigens in patients with symptoms of hypersensitivity. Clin. Chim. Acta.

[21] Stone M., Di Germanio C., Wright D.J., Sulaeman H., Dave H., Fink R.V., Notari E.P., Green V., Strauss D., Kessler D. (2022). Use of US blood donors for national serosurveillance of severe acute respiratory syndrome coronavirus 2 antibodies: Basis for an expanded national donor serosurveillance program. Clin. Infect. Dis..

[22] Bouza E., Almirante B., Garcia Rodriguez J., Garnacho-Montero J., Salavert M., Munoz P., Sanguinetti M. (2020). Biomarkers of fungal infection: Expert opinion on the current situation. Rev. Esp. Quimioter..

[23] Brown G.D., Denning D.W., Gow N.A., Levitz S.M., Netea M.G., White T.C. (2012). Hidden killers: Human fungal infections. Sci. Transl. Med..

[24] Lass-Flörl C., Kofler G., Kropshofer G., Hermans J., Kreczy A., Dierich M.P., Niederwieser D. (1998). In-vitro testing of susceptibility to amphotericin B is a reliable predictor of clinical outcome in invasive aspergillosis. J. Antimicrob. Chemother..

[25] Dannaoui E., Borel E., Persat F., Piens M.A., Picot S. (2000). Amphotericin B resistance of *Aspergillus terreus* in a murine model of disseminated aspergillosis. J. Med. Microbiol..

[26] Walsh T.J., Petraitis V., Petraitiene R., Field-Ridley A., Sutton D., Ghannoum M., Sein T., Schaufele R., Peter J., Bacher J. (2003). Experimental pulmonary aspergillosis due to *Aspergillus terreus*: Pathogenesis and treatment of an emerging fungal pathogen resistant to amphotericin B. J. Infect. Dis..

[27] Posch W., Blatzer M., Wilflingseder D., Lass-Flörl C. (2018). *Aspergillus terreus*: Novel lessons learned on amphotericin B resistance. Med. Mycol..

[28] Subhan M., Faryal R., Macreadie I. (2016). Exploitation of *Aspergillus terreus* for the production of natural statins. J. Fungi.

[29] Ruping M.J., Gerlach S., Fischer G., Lass-Flörl C., Hellmich M., Vehreschild J.J., Cornely O.A. (2011). Environmental and clinical epidemiology of *Aspergillus terreus*: Data from a prospective surveillance study. J. Hosp. Infect..

[30] Howard W.A., Gibson K.L., Dunn-Walters D.K. (2006). Antibody quality in old age. Rejuvenation Res..

[31] Chen W.H., Kozlovsky B.F., Effros R.B., Grubeck-Loebenstein B., Edelman R., Sztein M.B. (2009). Vaccination in the elderly: An immunological perspective. Trends Immunol..

[32] Page I.D., Richardson M.D., Denning D.W. (2016). Comparison of six *Aspergillus*-specific IgG assays for the diagnosis of chronic pulmonary aspergillosis (CPA). J. Infect..

[33] Lee M.R., Huang H.L., Chen L.C., Yang H.C., Ko J.C., Cheng M.H., Chong I.W., Lee L.N., Wang J.Y., Dimopoulos G. (2020). Seroprevalence of *Aspergillus* IgG and disease prevalence of chronic pulmonary aspergillosis in a country with intermediate burden of tuberculosis: A prospective observational study. Clin. Microbiol. Infect..

[34] Tuero I., Mohanram V., Musich T., Miller L., Vargas-Inchaustegui D.A., Demberg T., Venzon D., Kalisz I., Kalyanaraman V.S., Pal R. (2015). Mucosal B Cells are associated with delayed SIV acquisition in vaccinated female but not male rhesus macaques following SIVmac251 rectal challenge. PLoS Pathog..

[35] Mohanram V., Demberg T., Musich T., Tuero I., Vargas-Inchaustegui D.A., Miller-Novak L., Venzon D., Robert-Guroff M. (2016). B cell responses associated with vaccine-induced delayed SIVmac251 acquisition in female rhesus macaques. J. Immunol..

[36] Klein S.L., Flanagan K.L. (2016). Sex differences in immune responses. Nat. Rev. Immunol..

